# Risk factors for refractory BPPV: subtype-dependent association of serum 25-hydroxyvitamin D

**DOI:** 10.3389/fneur.2026.1838362

**Published:** 2026-06-03

**Authors:** Hye Ah Joo, Sun A. Han, Jin Kim, Il-Seok Park, Min Young Kwak

**Affiliations:** Department of Otorhinolaryngology-Head and Neck Surgery, Hallym University Dongtan Sacred Heart Hospital, Hallym University College of Medicine, Hwaseong-si, Gyeonggi-do, Republic of Korea

**Keywords:** 25-hydroxyvitamin D, benign paroxysmal positional vertigo, canalith repositioning procedures, dizziness, vertigo

## Abstract

Benign paroxysmal positional vertigo (BPPV) generally responds well to canalith repositioning procedures (CRPs), although some patients require repeated interventions. In this study, we evaluated refractory BPPV based on the number of CRP attempts required in 171 patients with acute BPPV and investigated clinical factors associated with treatment resistance, with particular emphasis on serum 25-hydroxyvitamin D [25(OH)D] levels and BPPV subtype. Kaplan–Meier analysis showed that cumulative resolution increased substantially during the first three CRP attempts but plateaued thereafter, indicating limited additional benefit beyond three sessions. Refractory BPPV was therefore defined as requiring three or more CRP attempts, and 47 patients (27.5%) met this criterion.

In the multivariable model without interaction terms, BPPV subtype and prior vestibular neuritis were the strongest predictors of treatment resistance, and lower serum 25(OH)D levels were independently associated with refractoriness. However, inclusion of an interaction term between 25(OH)D and subtype revealed a significant subtype-dependent effect, attenuating the main effect of 25(OH)D. Subgroup analysis demonstrated that lower 25(OH)D levels were associated with treatment resistance only in typical BPPV. These findings suggest that 25(OH)D may serve as a supplementary prognostic marker reflecting metabolic vulnerability of the otolith system, particularly in typical BPPV.

## 1 Introduction

Benign paroxysmal positional vertigo (BPPV) is the most common cause of acute vertigo and is generally attributed to displacement of otoconia from the utricular or saccular macula. Diagnosis is established using positional provocation tests that induce characteristic vertigo and nystagmus by eliciting otoconial movement within the affected canal. Treatment, similar in principle to diagnosis, consists of canalith repositioning procedures (CRPs) aimed at guiding the displaced otoconia back into the vestibule and out of the semicircular canal. Various repositioning maneuvers have been developed according to the involved canal, and these maneuvers are highly effective (1).

In clinical practice, challenging situations arise when patients present with atypical positional nystagmus or show inadequate response to CRPs (2). In most cases, the clinical presentation remains consistent with BPPV, leaving clinicians with limited options other than repeated CRPs.

Despite its clinical importance, the pathophysiological mechanisms underlying atypical or refractory BPPV remain poorly understood, and standardized management strategies have not been established. Various alternative treatments have been proposed, but their efficacy is limited (3–5), their effectiveness is often limited or accompanied by potential risks. Therefore, identifying objective clinical or metabolic factors associated with treatment refractoriness is of considerable importance (6).

The existing literature suggests that treatment resistance in BPPV is multifactorial, involving anatomical variations of the vestibular system, prior vestibular or cochlear dysfunction, and systemic metabolic or hormonal conditions that may compromise otoconial stability and post-CRP clearance (7–10). Structural vulnerability of macular sensory hair cells involved in otoconial formation and maintenance may also contribute to this process.

Another potential factor relates to the structural components of otoconial formation. Otoliths are biomineralized structures composed of calcium carbonate (CaCO_3_) crystals and an organic matrix of specific proteins, collagen, and proteoglycans. They are embedded in the maculae of the utricle and saccule (11). Their maintenance depends on the delicate regulation of the endolymphatic ionic environment and calcium concentrations (12–14). In this context, vitamin D plays an important role in calcium metabolism, and low serum 25-hydroxyvitamin D (25(OH)D) levels may disrupt ionic balance, leading to reduced otoconial stability and increased fragmentation Recent studies have primarily focused on the association between low bone mineral density (BMD) and reduced serum 25-hydroxyvitamin D (25(OH)D) levels with an increased risk of BPPV recurrence, as well as on the preventive effects of vitamin D supplementation and osteoporosis treatment on BPPV recurrence (15–20). However, the direct relationship between treatment refractoriness in BPPV and serum 25(OH)D levels has not been sufficiently investigated.

This study aimed to evaluate BPPV typicality and resistance to canalith repositioning procedures (CRPs) in adults with acute idiopathic BPPV, and to identify clinical and metabolic factors associated with treatment refractoriness. In particular, we focused on serum vitamin D status—previously investigated mainly in relation to BPPV recurrence—and examined its association with treatment resistance in an integrated model alongside other established clinical risk factors, to identify independent predictors of poor CRP responsiveness.

## 2 Materials and methods

### 2.1 Participants, diagnostic classification, and treatment protocol

We conducted a retrospective cohort study evaluating the treatment course of 171 patients who presented with acute positional vertigo. Patients with positional nystagmus attributable to central lesions were excluded, and the study population was restricted to cases in which vertigo was solely explained by peripheral BPPV. Patients with secondary BPPV, including traumatic or postoperative BPPV and cases in which Meniere's disease was considered the primary cause of vertigo, were excluded. Cases with incomplete diagnostic evaluation or follow-up and those presenting more than 1 month after symptom onset were excluded.

Clinical variables analyzed comprised the timing of BPPV onset, onset-to-visit interval, number of prior BPPV episodes, BPPV subtype, serum 25-hydroxyvitamin D [25(OH)D] levels, previous history of vestibular neuropathy or sudden sensorineural hearing loss (SSNHL). Additional relevant medical comorbidities were included in the analysis, including diabetes mellitus, hypertension, cerebrovascular disease, osteoporosis, and vitamin D deficiency. Osteoporosis was identified through a retrospective review of electronic medical records (EMR). The diagnosis was primarily based on a documented clinical history of osteoporosis or prior bone mineral density (BMD) measurements using Dual-energy X-ray Absorptiometry (DEXA), following the World Health Organization (WHO) diagnostic criteria (T-score ≤ -2.5).

**Figure 1:**
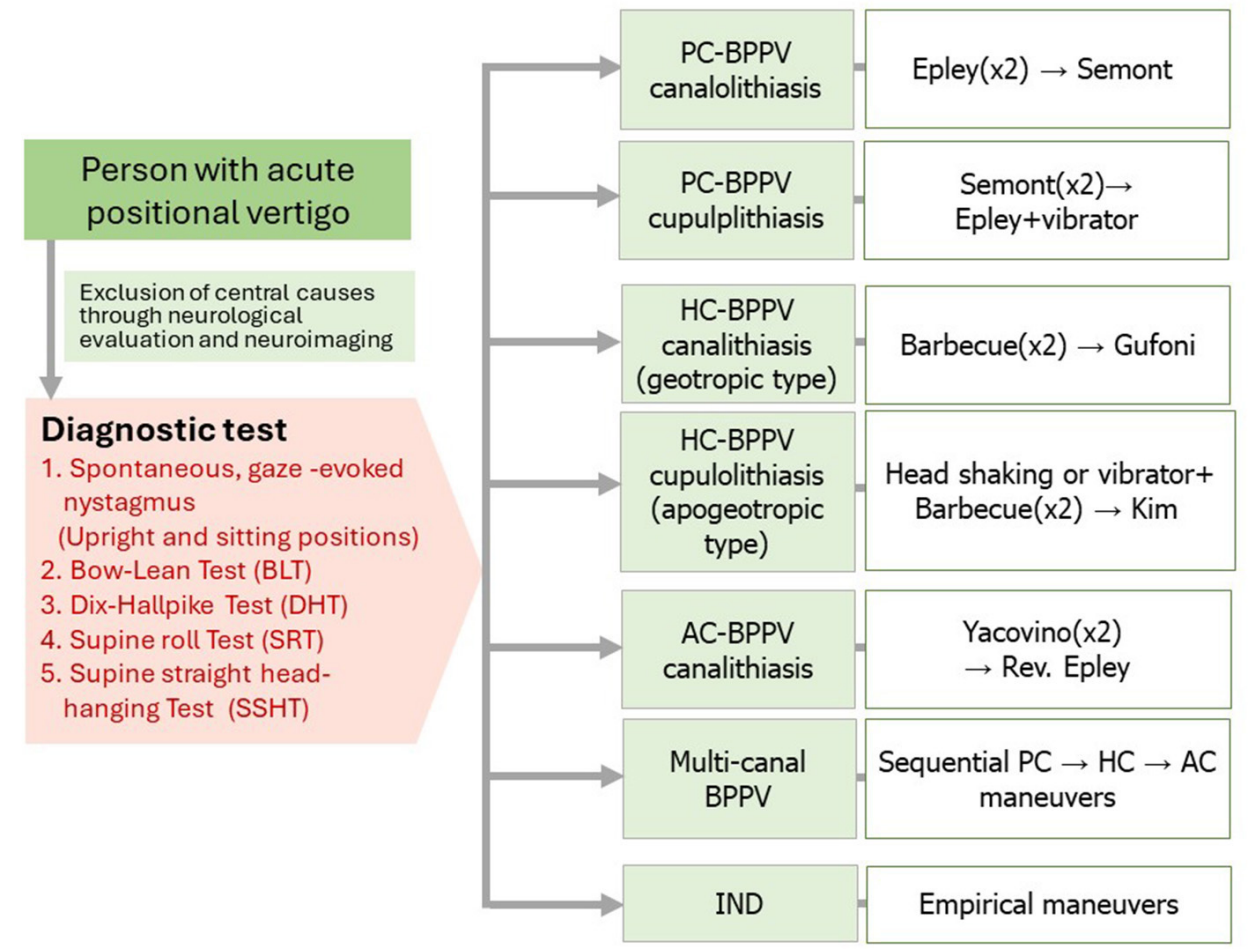
Diagnostic workflow and subtype-specific treatment algorithm for acute BPPV. Following exclusion of central causes, patients were classified into mechanical subtypes based on positional nystagmus patterns and treated with appropriate canalith repositioning procedures. Standard positional maneuvers include the Bow–Lean Test (BLT), Dix–Hallpike Test (DHT), Supine Roll Test (SRT), and Straight Head-Hanging Test (SSHT).

Figure 1 summarizes the standardized diagnostic classification used in this cohort. To ensure diagnostic accuracy and identify potential multi-canal involvement, all patients underwent a full battery of positional tests in a fixed sequence.

After subtype identification based on positional nystagmus characteristics, patients underwent subtype-specific CRPs tailored to the involved semicircular canal and presumed otolith mechanism. BPPV subtype classification followed the diagnostic criteria for BPPV proposed by the Bárány Society in 2017 (21), according to which “*typical BPPV*” includes posterior canal canalolithiasis, horizontal canal canalolithiasis, and horizontal canal cupulolithiasis.

In this study, all cases not classified into the three predefined typical BPPV subtypes were operationally defined as atypical BPPV. This category included less common and debated entities such as anterior canal canalolithiasis, posterior canal cupulolithiasis, and multi-canal BPPV, as well as the “light cupula” and “heavy cupula” variants, identified by persistent apogeotropic or geotropic positional nystagmus during the head-roll test. All cases were classified according to established diagnostic criteria based on the pattern, duration, and latency of nystagmus observed during specific positional tests, and standard clinical terminology was applied as reported in the literature.

“*Refractory BPPV*” was defined as persistent positional vertigo and nystagmus despite three appropriately performed canal-specific CRPs (6, 22). Under the study protocol, CRPs were administered alternately by two clinicians at 2–3-day intervals, and were continued beyond three sessions when clinically indicated, with some patients undergoing more than five treatment sessions.

All treatment procedures were performed in accordance with the standardized algorithm shown in Figure 1. For typical posterior canal BPPV, two consecutive Epley maneuvers were performed, followed by the Semont maneuver in cases of treatment failure. Typical horizontal canal BPPV was managed with repeated Barbecue maneuvers, with the Gufoni maneuver reserved for refractory cases. In horizontal canal cupulolithiasis, treatment included head-shaking or mastoid vibration in combination with repositioning maneuvers, followed by the Kim maneuver as appropriate. Posterior canal cupulolithiasis was primarily managed using the Semont maneuver or an Epley maneuver with adjunctive mastoid vibration. Anterior canal BPPV was initially treated with the Yacovino maneuver, with the reverse Epley maneuver reserved for cases with repeated therapeutic failure.

In cases of multi-canal BPPV, the posterior canal was prioritized for initial treatment, followed sequentially by the horizontal and anterior canals. When BPPV was clinically suspected, the exact canal involvement was not always clearly identified based on nystagmus direction or components. In such cases, empiric canal-specific CRPs were performed at the clinician's discretion.

In summary, patients presenting with acute positional vertigo and diagnosed with BPPV were classified into typical and atypical BPPV, and standardized diagnostic tests and treatment protocols were applied. Treatment response was evaluated based on the cumulative number of canalith repositioning procedures (CRPs), and the distribution of refractory BPPV was assessed. In addition, clinical factors associated with CRP failure were analyzed, with particular emphasis on the association with serum vitamin D levels.

### 2.2 Statistical analysis

Statistical analyses were performed using SPSS 29 (IBM Corp., Armonk, NY, USA). Baseline characteristics were summarized using mean ± standard deviation (SD) for continuous variables and counts with percentages (%) for categorical variables. A Kaplan–Meier survival analysis was conducted to estimate cumulative probabilities of symptom resolution according to the number of repeated CRP sessions. Ordinal logistic regression analysis was performed to identify factors associated with the number of CRP attempts required for symptom resolution. Sex and age were included as baseline covariates, and variables that were significant in univariate analyses or considered clinically relevant were entered into the multivariable model. In addition, to evaluate the interaction effect between serum vitamin D levels and BPPV subtype, an ordinal logistic regression analysis including an interaction term between the two variables was performed.

## 3 Results

### 3.1 Baseline characteristics and canalith repositioning outcomes in patients with acute BPPV

Baseline clinical characteristics of the 171 patients with acute BPPV enrolled in this study are presented in Table 1. A total of 171 patients were included in the analysis, with a mean age of 59.8 ± 12.9 years; 55.0% were female. A prior history of sudden sensorineural hearing loss and vestibular neuropathy was identified in 19.2% and 19.4% of patients, respectively. The mean serum 25(OH)D level was 27.0 ± 12.7 ng/ml, and the mean onset-to-diagnosis interval was 7.0 ± 11.0 days. Typical BPPV accounted for 63.7% of cases, most commonly posterior canal canalolithiasis (32.7%), whereas 36.3% were classified as atypical subtypes.

**Table 1:** Baseline clinical characteristics of all idiopathic BPPV patients included in the study (*n*= 171).

Variables	Total (*n* = 171)
Age (years), mean ± SD	59.84 ± 12.90 [21.0–87.0]
Sex, M: F	77 (45.0%): 94 (55.0%)
BPPV side R: L: B: IND	84 (49.1%): 77 (45.0%): 3 (1.8%): 7 (4.1%)
Number of prior BPPV diagnoses	
0 (none), *n*	75/170 (44.1%)
1–2 prior BPPV diagnosis, *n*	58/170 (34.1%)
≥3 prior BPPV diagnoses, *n*	37/170 (21.7%)
History of SSNHL, *n* (%)	33/171 (19.2%)
Past vestibular function impairment, *n* (%)	33/170 (19.4%)
HTN, *n* (%)	39/171 (22.8%)
DM, *n* (%)	19/171 (11.1%)
CVD, *n* (%)	17/171 (9.9%)
Osteoporosis, *n* (%)	12/85 (14.1%)
25(OH)D (ng/ml), mean ± SD	27.0 ± 12.7 [6.7–65.0]
Onset-to-diagnosis interval (days), mean ± SD	7.01 ± 11.01 [0–21]
BPPV subtypes	
**Typical (*****n*****, %)**	**109/171 (63.7%)**
PC canalolithiasis	56 (32.7%)
HC canalolithiasis	34 (19.9%)
HC cupulolithiasis	19 (11.1%)
**Atypical (*****n*****, %)**	**62/171 (36.3%)**
AC canalolithiasis	8 (4.7%)
PC cupulolithiasis	14 (8.2%)
HC light cupula	11 (6.4%)
HC heavy cupula	10 (5.8%)
Multicanal	14 (8.2%)
IND	5 (2.9%)
**Total CRP sessions (mean** **±SD)**	**1.33** **±1.53**
1^*^	83/171 (48.5%)
2	24/171 (14.0%)
3	17/171 (9.9%)
4	23/171 (13.5%)
≥5	24/171 (14.0%)

SSNHL, idiopathic sudden sensorineural hearing loss; HTN, hypertension; DM, diabetes mellitus; CVD, cerebrovascular disease; IND, indeterminate; PC, posterior canal; HC, horizontal canal; AC, anterior canal; CRP, canal repositioning procedure.^*^Failure was defined as persistence of vertigo and positional nystagmus after each CRP session.

The mean number of CRP sessions required was 1.33 ± 1.53. Approximately 48.5% of patients achieved symptom resolution without CRP failure, whereas 27.5% required three or more treatment attempts, meeting the predefined criteria for treatment-resistant BPPV.

### 3.2 Differential cumulative CRP response between typical and atypical BPPV

**Figure 2:**
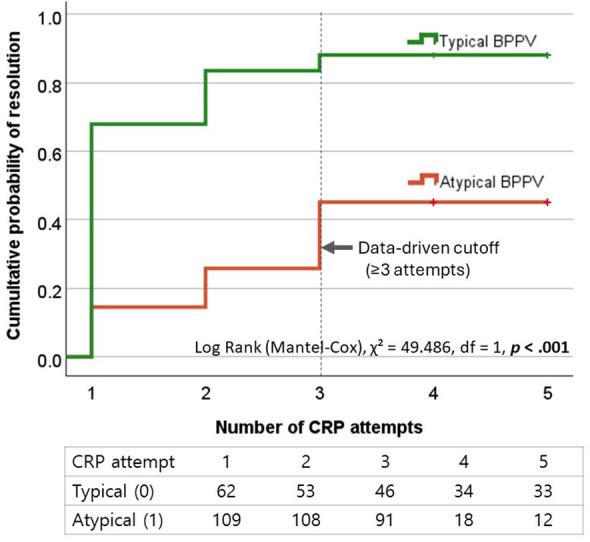
Differential cumulative CRP resolution according to BPPV subtype. Kaplan–Meier curves show the cumulative probability of symptom resolution according to the number of canalith repositioning procedure (CRP) attempts in patients with typical and atypical BPPV. Typical BPPV demonstrated a significantly steeper cumulative response compared with atypical BPPV (log-rank test, χ^2^ = 49.486, df = 1, *p* < 0.001). By the third CRP attempt, cumulative resolution reached approximately 88–90% in typical BPPV, whereas it remained below 50% in atypical cases. The dashed vertical line indicates the data-driven cutoff (≥3 attempts) used to define treatment-resistant BPPV. Numbers at risk for each CRP attempt are shown below the plot.

Kaplan–Meier analysis demonstrated a significant difference in cumulative CRP success between typical and atypical BPPV subtypes (log-rank test, χ^2^ = 49.486, df = 1, p < 0.001, Figure 2). The typical subtype showed a steeper cumulative response slope compared with the atypical subtype. By the third attempt, cumulative resolution reached approximately 88%−90% in typical BPPV but remained below 50% in atypical BPPV, after which both curves demonstrated a plateau pattern with limited additional gain. However, in the atypical group, a measurable increase in resolution probability was observed at the third maneuver compared with the second. These findings imply that at least three CRP attempts may be warranted before considering the condition refractory, particularly in atypical BPPV.

### 3.3 Factors influencing repeated CRP failure

In the multivariable model without interaction terms (Model 1, Table 2), BPPV subtype and prior vestibular pathology (VP) were the strongest predictors of refractoriness. BPPV subtype demonstrated the largest effect size (OR 10.99, 95% CI 5.58–21.70, *p* < 0.001), followed by prior vestibular pathology (OR 0.240, 95% CI 0.109–0.531, *p* < 0.001). Serum vitamin D level was also significantly associated with refractoriness (OR 0.971, 95% CI 0.945–0.996, *p* = 0.022), indicating that lower vitamin D levels were associated with increased CRP requirement.

**Table 2:** Ordinal logistic regression models and forest plot of odds ratios for CRP failure frequency.

Variable	Model 1 (No interaction) OR (95% CI)	*p*-value	Model 2 (With interaction) OR (95% CI)	*p-*value	Forest plot of odds ratios for CRP failure frequency (Model 2: with interaction)
Age	1.014 (0.990–1.039)	0.243	1.011 (0.987–1.036)	0.383	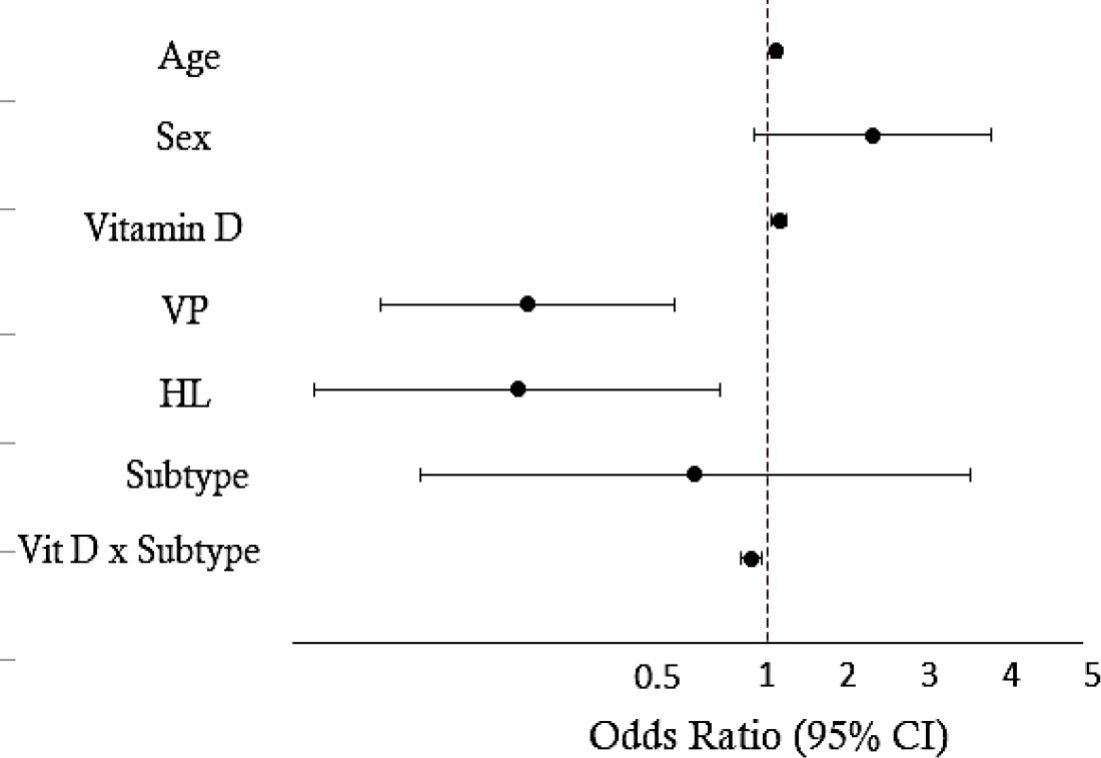
Sex	1.392 (0.747–2.599)	0.298	1.721 (0.900–3.292)	0.100	
Vitamin D	0.971 (0.945–0.996)	0.022^*^	1.031 (0.991–1.072)	0.137	
VP	0.240 (0.109–0.531)	< 0.001^***^	0.261 (0.117–0.584)	0.001^**^	
HL	0.398 (0.139–1.135)	0.085	0.248 (0.082–0.749)	0.013^*^	
Subtype	10.99 (5.58–21.70)	< 0.001^***^	0.653 (0.146–2.918)	0.577	
VitD × subtype	—	—	0.887 (0.836–0.941)	< 0.001^***^	

Ordinal logistic regression (proportional odds model, logit link) was performed to evaluate factors associated with increasing CRP failure frequency (0–4 attempts). Model 1 adjusted for age, sex, VP history, HL, and BPPV subtype. Model 2 additionally included the interaction term between vitamin D level and BPPV subtype. Odds ratios (OR) represent cumulative odds of higher CRP requirement. Sex: 1 = female; VP, presence of prior vestibular pathology; HL, presence of hearing loss; Subtype, 1 = typical BPPV. ORs represent cumulative odds of increasing CRP failure frequency (0–4 attempts). ^†^SSNHL: prior sudden idiopathic sensorineural hearing loss ‡Past vestibular neuropathy: history of vestibular function impairment CRP, canalith repositioning procedure; OR: odds ratio; CI: confidence interval. ^*^p < 0.05; ^**^p < 0.01; ^***^p < 0.001.

### 3.4 Interaction between vitamin D and BPPV subtype

Given the dominant effect of BPPV subtype, we additionally performed an interaction analysis between vitamin D level and subtype (Model 2, Table 2). When the interaction term between vitamin D and BPPV subtype was introduced, a significant interaction effect was observed (OR 0.887, 95% CI 0.836–0.941, *p* < 0.001). In this model, the main effects of vitamin D and subtype were attenuated, indicating that the association between vitamin D and CRP failure severity was dependent on subtype. Specifically, vitamin D was not significantly associated with CRP severity in atypical BPPV. However, in typical BPPV, higher vitamin D levels were associated with a lower likelihood of requiring multiple CRP attempts. These findings suggest that the effect of vitamin D is not uniform across mechanical subtypes but is primarily evident in typical BPPV.

**Figure 3:**
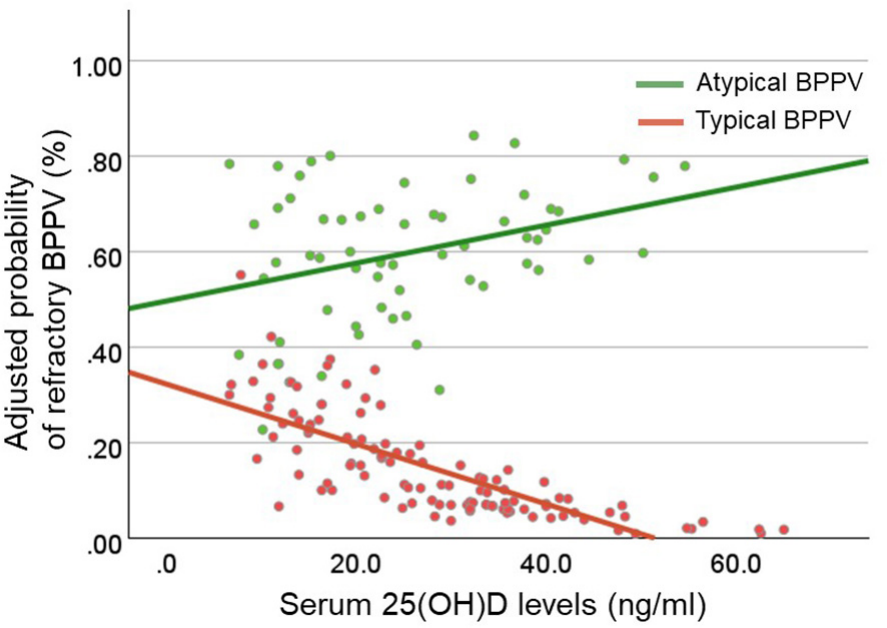
Subtype-dependent association between serum 25(OH)D levels and refractory BPPV probability. Interaction plot showing adjusted probability of refractory BPPV according to serum 25(OH)D levels by subtype. A significant divergence in slopes demonstrates that the effect of vitamin D differs between typical and atypical BPPV. In typical BPPV, increasing vitamin D levels were associated with a progressive reduction in the probability of refractory disease. In contrast, no meaningful protective association was observed in atypical BPPV.

Figure 3 illustrates the significant interaction between serum 25(OH)D levels and BPPV subtype (*p* < 0.001). In typical BPPV, increasing vitamin D levels were associated with a marked reduction in the adjusted probability of refractory BPPV. Conversely, in atypical BPPV, the probability of refractory disease was not meaningfully reduced with increasing vitamin D levels. The divergence of regression slopes confirms that the effect of vitamin D on CRP failure severity differs according to mechanical subtype.

## 4 Discussion

### 4.1 Key findings of this study

The present study provides several clinically meaningful insights into treatment-resistant BPPV. First, refractory BPPV was not uncommon in this cohort, affecting approximately one-quarter of the patients. It should be noted that this finding may be influenced by referral bias; as an academic referral center, our institution frequently manages complex and atypical BPPV cases that are referred from primary clinics due to their challenging nature.

Second, among all evaluated variables, the BPPV subtype emerged as the strongest determinant of repeated CRP failure. Specifically, patients with atypical subtypes demonstrated substantially poorer cumulative CRP responses compared to those with typical BPPV.

Third, although lower serum 25(OH)D levels were initially associated with increased CRP requirements in the main multivariable model, this relationship was not uniform across subtypes. The significant interaction between vitamin D level and BPPV subtype indicates that the prognostic effect of vitamin D is subtype-dependent rather than universal. Specifically, vitamin D demonstrated a protective association in typical BPPV but not in atypical presentations. This finding suggests that metabolic factors may influence canalith dynamics differently depending on the underlying mechanical and pathophysiological substrate.

Fourth, prior vestibular pathology and inner ear disease history were independently associated with treatment refractoriness, supporting the hypothesis that pre-existing vestibular vulnerability may impair otolith repositioning efficiency or central compensatory mechanisms.

Collectively, these findings indicate that treatment-resistant BPPV reflects an interaction between mechanical subtype and patient-specific vestibular vulnerability rather than vitamin D deficiency alone. Accordingly, prediction of CRP outcomes should integrate phenotypic classification with inner ear functional status and systemic metabolic factors.

### 4.2 Refractory BPPV

In previous retrospective studies, refractory or intractable BPPV has commonly been defined as the absence of symptom resolution after two to five canalith repositioning procedures (CRPs) (23–25). However, no universally accepted maneuver-based threshold exists, and most studies rely on pragmatic definitions such as persistence despite repeated repositioning.

In our cohort analysis, subtype-stratified analysis showed that cumulative resolution plateaued after three CRPs. This finding suggests that at least three repositioning attempts are reasonable before labeling a case as refractory, particularly in atypical BPPV, where repeated maneuvers may provide incremental benefit.

Proposed mechanisms of refractoriness—including canalith impaction (26), canalith jam (27), or immobilized otoconia—remain hypothetical and lack direct histopathological confirmation. Likewise, persistent horizontal positional nystagmus patterns described as “light cupula” or “heavy cupula” represent descriptive clinical constructs rather than pathologically established disease entities (28). Some investigators argue that these variants should not be regarded as true BPPV. Nevertheless, considering their clinical management with CRP, it may be prudent to interpret them as mechanical phenotypes within the spectrum of BPPV rather than as completely separate disease entities.

Several factors have been associated with poor CRP response, including advanced age, post-traumatic BPPV, and secondary forms related to viral inner ear disorders (1, 29). Another important finding of this study was that the history of vestibular dysfunction and hearing loss was significantly associated with increased CRP failure. This observation is consistent with previous reports and suggests that vulnerability of both the vestibular and cochlear systems may influence treatment response in BPPV. A prior history of inner ear damage may affect the formation and structural stability of otoconia, rendering them more prone to detachment or fragmentation. This, in turn, may contribute to persistent symptoms or reduced responsiveness to repositioning maneuvers. Previous studies have reported that patients with a history of vestibular disorders, such as vestibular neuritis, may have poorer BPPV outcomes. In addition, several studies have suggested that sudden sensorineural hearing loss may be associated with an increased incidence of BPPV or reduced treatment response (30). Taken together, these findings suggest that dysfunction of the vestibular and cochlear systems may reflect an underlying inner ear vulnerability that contributes to treatment refractoriness in BPPV. This perspective may have important implications for prognostic assessment and the development of individualized treatment strategies.

According to the previous studies, metabolic conditions such as vitamin D deficiency have been more consistently linked to recurrence than to treatment failure (31). In our study, lower serum 25(OH)D levels were associated with CRP refractoriness in the multivariable model. However, this effect was attenuated after accounting for BPPV subtype, suggesting a subtype-dependent interaction. These findings indicate that vitamin D deficiency alone may not directly determine treatment resistance but rather reflects a subtype-specific association predominantly observed in typical BPPV.

Collectively, our findings support a multidimensional model of BPPV refractoriness in which BPPV subtype plays a dominant role, while metabolic factors such as vitamin D may exert a subtype-specific modulatory effect.

### 4.3 Previous studies examining the relationship between 25(OH)D levels and BPPV

Most prior studies examining serum 25(OH)D in BPPV have focused on residual dizziness after successful CRP, recovery quality, or long-term recurrence prevention. Only a single prior study has examined predictors of treatment-resistant BPPV (32).

Previous studies evaluating the association between serum 25(OH)D levels and residual dizziness (RD) after CRP have largely prioritized quality of recovery and long-term outcomes. RD refers to persistent subjective dizziness or imbalance despite resolution of positional nystagmus and negative positional tests after successful CRP. Wu Y reported that residual dizziness was present in 41.5% of PC BPPV patients following clinically successful CRP, and that lower 25(OH)D levels were associated with poorer subjective recovery (*p* = 0.001), suggesting a potential relationship between 25(OH)D insufficiency and delayed symptomatic resolution (33). Similarly, Zhou X observed that more severe 25(OH)D deficiency corr-lated with a higher incidence of RD, greater dizziness handicap inventory (DHI) scores, and abnormal cVEMP responses, proposing that 25(OH)D status may contribute to risk stratification for residual symptomatic burden when combined with vestibular vulnerability indicators (34).

Another axis of investigation includes longitudinal studies assessing clinical indices after 25(OH)D supplementation. Gu X and Sheikhzadeh M both described trends toward reduced vertigo intensity, improved balance measures, and lower DHI scores following 25(OH)D supplementation (35, 36). However, the benefit of 25(OH)D in this context is more plausibly aligned with maintaining utricular macular stability and reducing the propensity for additional otoconial detachment or recurrence, rather than functioning as a direct intervention for already displaced canaliths. This interpretation may be more congruent from a theoretical and biological standpoint.

Another line of research has focused on the preventive role of 25(OH)D in BPPV recurrence. A multicenter randomized controlled trial demonstrated that vitamin D supplementation after successful CRP significantly reduced BPPV recurrence in patients with subnormal baseline levels. Subsequent systematic reviews and meta-analyses have generally supported a protective effect of vitamin D supplementation on recurrence risk, although effect sizes vary across studies (30, 37, 38).

Temirbekov and Sari (32), including 122 patients—has directly evaluated predictors of treatment-resistant or intractable BPPV defined by the need for ≥3 repositioning maneuvers. In that analysis, male sex (OR ≈ 3), comorbid conditions, particularly hypertension, and concurrent betahistine exposure were significantly associated with a greater likelihood of requiring ≥3 maneuvers, whereas serum 25(OH)D levels were not, leading the authors to conclude that 25(OH)D is not an independent prognostic determinant for intractable or CRP-resistant BPPV.

In contrast, our cohort showed a lower proportion of patients requiring ≥3 CRP attempts (47/171, 27.5%) compared with the 44.3% (54/122) reported by Temirbekov et al. While previous literature generally estimates refractory BPPV to account for approximately 10–20% of idiopathic cases, variations in reported rates may reflect differences in patient selection, clinical definitions, or case heterogeneity across studies. Additionally, serum 25(OH)D in the Temirbekov study was categorized using a binary cutoff (< 19 vs. ≥20 ng/ml). Although this approach provides practical clinical interpretability, such categorization may reduce statistical sensitivity to detect graded or subtype-specific prognostic associations.

### 4.4 Interpretation of the association observed only in typical BPPV

In the present study, serum 25(OH)D level was not an independent predictor in the overall population; however, a significant association with treatment refractoriness was observed specifically in the typical BPPV subgroup. This finding suggests that the prognostic relevance of Vitamin D may not be uniform across all BPPV cases but may be more pronounced in subtypes with a well-defined mechanical pathophysiology.

Typical BPPV is primarily characterized by canalolithiasis, in which displaced otoconia move within the semicircular canals. The formation and structural stability of these otoconia are intimately linked to calcium metabolism; specifically, 25(OH)D plays a pivotal role in maintaining calcium homeostasis and ensuring otoconial integrity within the inner ear.

Previous studies have confirmed the presence of vitamin D receptors (VDR) within the otolith organs, suggesting that vitamin D deficiency—similar to its effects in osteoporosis models—can lead to a reduction in otoconial mass and density or induce structural abnormalities. Such alterations in physical properties, particularly decreased density and increased fragmentation, may fundamentally compromise the hydrodynamic transport efficiency of the otoconia. Consequently, rather than simply increasing the likelihood of displacement, these physical changes may hinder the smooth movement of otoconial debris through the endolymph during therapeutic maneuvers. This provides a plausible mechanical basis for the reduced responsiveness to Canalith Repositioning Procedures (CRP) observed specifically in patients with typical BPPV.

In contrast, atypical BPPV encompasses heterogeneous entities, including light or heavy cupula, multi-canal involvement, and cases associated with concurrent vestibular pathology. Such variability suggests that mechanisms beyond simple disturbances in calcium metabolism may be involved. Under these conditions, the impact of Vitamin D may be attenuated or overshadowed by other pathophysiologic factors. Notably, an opposite trend was observed in atypical BPPV, although this should be interpreted cautiously given the heterogeneity of this group. This interpretation is consistent with our finding that the association between Vitamin D level and treatment refractoriness differed according to subtype.

Taken together, our results indicate that Vitamin D may not function as a universal prognostic marker in BPPV but may have greater biological and clinical relevance in typical mechanical subtypes.

## 5 Limitation and strength

This study has several limitations. First, its retrospective, single-center design in a university-affiliated referral center may introduce selection and referral bias, potentially overrepresenting more complex or treatment-resistant cases and limiting generalizability. The retrospective nature also raises the possibility of misclassification, particularly in atypical BPPV subtypes. Second, the definition of refractory BPPV as requiring ≥3 CRPs, although data-driven, is not universally standardized and may limit comparability with prior studies. Third, grouping heterogeneous conditions under “atypical BPPV” may obscure subtype-specific effects and complicate interpretation of the observed interaction with Vitamin D. For instance, there is a potential clinical overlap between patients exhibiting atypical positional nystagmus and Vestibular Migraine (VM). Existing literature has reported that VM can induce atypical nystagmus mimicking BPPV or that the two conditions may coexist. While the present study focused on the mechanical and physical aspects of BPPV in relation to Vitamin D, future research investigating the interactions between BPPV—particularly in cases with atypical positional nystagmus—and concomitant factors such as VM would be highly valuable. Clarifying how these specific factors influence otoconial stability and treatment responsiveness will provide a more profound academic foundation for understanding the complex pathophysiology of refractory BPPV.

Fourth, serum 25(OH)D was measured at a single time point without accounting for longitudinal variation, supplementation, or seasonal effects, limiting causal interpretation. Finally, the number of CRPs required, used as the primary outcome, may be influenced by clinician decision-making and procedural variability rather than solely reflecting underlying pathophysiology.

Nevertheless, by defining treatment resistance based on consecutive CRP attempts (≥3), this study provides a clinically relevant perspective distinct from the predominantly recurrence-focused literature. Notably, the association between serum 25(OH)D levels and treatment resistance was observed only in the typical BPPV subtype, suggesting that Vitamin D may not serve as a universal marker across all BPPV cases but rather as a subtype-specific prognostic indicator.

## 6 Conclusion

In this cohort, treatment-resistant BPPV was defined as requiring ≥3 canalith repositioning procedures, and its clinical characteristics were systematically evaluated. BPPV subtype remained the strongest predictor of poor CRP response. Although serum 25(OH)D level was not an independent predictor in the overall population, a significant association with treatment resistance was observed specifically in the typical BPPV subtype.

These findings suggest that the prognostic relevance of Vitamin D may not be uniform across all BPPV cases but may have greater clinical significance within specific mechanical subtypes. Further multicenter and prospective studies are warranted to validate these subtype-specific associations and to refine risk stratification strategies for treatment-resistant BPPV.

## Data Availability

The raw data supporting the conclusions of this article will be made available by the authors, without undue reservation.
